# Clinical Relevance of Digital Rectal Examination in Men With Low and Very Low Prostate-Specific Antigen (PSA): Reassessing Its Diagnostic Value in the MRI Era and in Light of Modern Guidelines

**DOI:** 10.7759/cureus.99145

**Published:** 2025-12-13

**Authors:** Toni Franz, Jens-Uwe Stolzenburg

**Affiliations:** 1 Department of Urology, University of Leipzig, Leipzig, DEU

**Keywords:** clinically significant prostate cancer, digital rectal examination, low psa, prostate cancer, prostate cancer screening

## Abstract

Background/aim

Digital rectal examination (DRE) has declined in importance as a primary screening tool for prostate cancer (PCa), yet it remains relevant as a selective clinical assessment. This study aimed to evaluate the residual diagnostic value of DRE in men with low prostate-specific antigen levels (PSA < 4 ng/mL), who would otherwise not undergo further diagnostic evaluation, and to identify cases in which PCa was detected primarily on the basis of an abnormal DRE finding.

Patients and methods

We conducted a retrospective analysis of a prospectively maintained, single-center database comprising all consecutive MRI-ultrasound fusion-guided prostate biopsies performed between August 2014 and September 2024 at a university high-volume center (n = 1,300). Men were eligible if they presented with elevated PSA and/or a suspicious DRE, with abnormal findings confirmed by a second senior urologist. For the present analysis, all patients with PSA < 4 ng/mL (Group A, n = 114) were selected and further stratified into two subgroups: PSA 3-4 ng/mL (Group B, n = 57) and PSA < 3 ng/mL (Group C, n = 57). Primary endpoints were the detection of overall PCa and clinically significant PCa (csPCa; International Society of Urological Pathology (ISUP) grade group ≥ 2). In addition, we evaluated the proportion of tumors associated with a positive DRE.

Results

No significant differences were observed between the three subgroups in the detection rates of overall PCa (64/114 vs. 31/57 vs. 33/57; p = 0.85) or csPCa (29/64 vs. 15/57 vs. 14/57; p = 1.00). However, DRE-associated PCa detection was significantly more frequent in the PSA < 3 ng/mL subgroup than between 3 and 4 ng/mL (24.6% vs. 8.8%; p = 0.044), with no corresponding difference in DRE-detected csPCa (p = 0.32). Among all DRE-positive patients with PSA < 4 ng/mL, PCa and csPCa detection rates were 67.9% and 35.7%, respectively, without significant differences between PSA strata (both p = 0.63). Without DRE, 16.8% of PCas (19/114) would not have been detected, including 10 clinically significant tumors (8.8%). Histopathological analysis showed predominantly low-grade tumors (peak ISUP 1), with fewer csPCa cases.

Conclusion

In men with PSA < 4 ng/mL, the diagnostic yield of DRE was limited, yet a clearly abnormal DRE remained a meaningful indicator of PCa and identified a small but relevant proportion of clinically significant tumors that would have been missed by PSA- and MRI-driven pathways alone. While DRE no longer serves as a screening tool, our findings support its selective use in the diagnostic work-up, where suspicious palpatory findings continue to warrant further evaluation.

## Introduction

Prostate cancer (PCa) remains one of the most prevalent malignancies among men worldwide, and early detection is essential for optimizing treatment outcomes and reducing disease-specific mortality [1-3]. Historically, digital rectal examination (DRE) played a central role in the early detection of PCa, as it could identify palpable abnormalities suggestive of malignancy of the prostate gland at the bedside. Due to its simplicity, immediate applicability, and lack of resource dependency, DRE was deeply embedded in routine urologic practice before the introduction of prostate-specific antigen (PSA) testing in the late 1980s [4].

However, with the development of more sensitive and specific diagnostic modalities, its importance in routine screening has declined. PSA‐based screening programs and especially multiparametric MRI (mpMRI) have markedly improved both the detection of clinically significant PCa (csPCa) and the reduction of unnecessary biopsies by enabling better risk stratification and targeted sampling [5-8]. Recent evidence indicates that DRE alone has limited sensitivity, particularly for anterior or small-volume tumors, and contributes only marginally to risk assessment when PSA and imaging findings are already available [9-11].

Reflecting this evolution, contemporary clinical guidelines clearly differentiate between screening and diagnostic evaluation. Both the European Association of Urology (EAU) and the American Urological Association (AUA) guidelines, as well as current German recommendations, no longer endorse DRE as a stand-alone screening tool due to its low sensitivity and minimal incremental benefit over PSA testing [5-7]. In particular, DRE contributes only minimal additional diagnostic value when PSA levels and mpMRI findings are available, especially in cases involving small or anterior tumors. However, in the diagnostic evaluation of patients with suspected PCa (e.g., elevated PSA), the EAU still recommends performing DRE because abnormal findings increase the likelihood of clinically significant disease and may guide biopsy targeting. Thus, within the modern diagnostic pathway, the role of DRE has shifted from a primary screening measure to a selective clinical assessment tool. While DRE retains value in selected clinical contexts, modern screening strategies increasingly rely on biomarker-based and imaging-driven approaches to improve diagnostic accuracy and minimize overdiagnosis.

The present study analyzes a large, real-world cohort of over 1,300 patients assessed across a 10-year period. Its primary objective is to quantify PCs that would have been missed without DRE by identifying patients in whom cancer was detected solely through a positive DRE, independent of PSA elevation or imaging findings. This subgroup is of particular clinical relevance, as recent national and international guideline recommendations increasingly de-emphasize the use of DRE in the context of primary screening.

By examining outcomes in patients whose cancer would potentially have remained undetected under contemporary diagnostic pathways, the study aims to clarify the residual diagnostic value of DRE and to contribute evidence to the ongoing discussion regarding its appropriate role in modern PCa detection strategies.

## Materials and methods

Study design and population

This retrospective analysis was based on a prospectively maintained, single-center database from the Department of Urology, University of Leipzig, Leipzig, Germany, including all consecutive MRI-ultrasound fusion-guided prostate biopsies performed since 2014. Men were eligible if they presented with elevated serum PSA and/or a suspicious DRE. In cases of a suspicious DRE finding, a confirmatory cross-check was performed by a second senior urologist. DRE criteria included assessment of prostate size, symmetry, consistency (soft, firm, or hard), surface regularity, presence of nodules, delineation of prostate borders, integrity of the median sulcus, tenderness, mobility versus fixation, and concurrent rectal abnormalities.

mpMRI was acquired using a standardized protocol comprising T2-weighted, diffusion-weighted (DWI), and dynamic contrast-enhanced (DCE) sequences after intravenous contrast administration, in accordance with Prostate Imaging-Reporting and Data System (PI-RADS) recommendations [8]. All examinations were evaluated by board-certified radiologists with more than 10 years of dedicated experience in prostate MRI, and lesions were reported according to PI-RADS v2.1 criteria [8]. In line with current standards, DWI served as the dominant sequence for peripheral zone lesions, while T2-weighted imaging was the primary sequence for transition zone assessment.

Between August 2014 and September 2024, a total of 1,300 men underwent fusion biopsy under local anesthesia with single-dose antibiotic prophylaxis. The biopsy protocol included systematic 12-core sampling supplemented by targeted cores from MRI-identified lesions.

**Figure 1:**
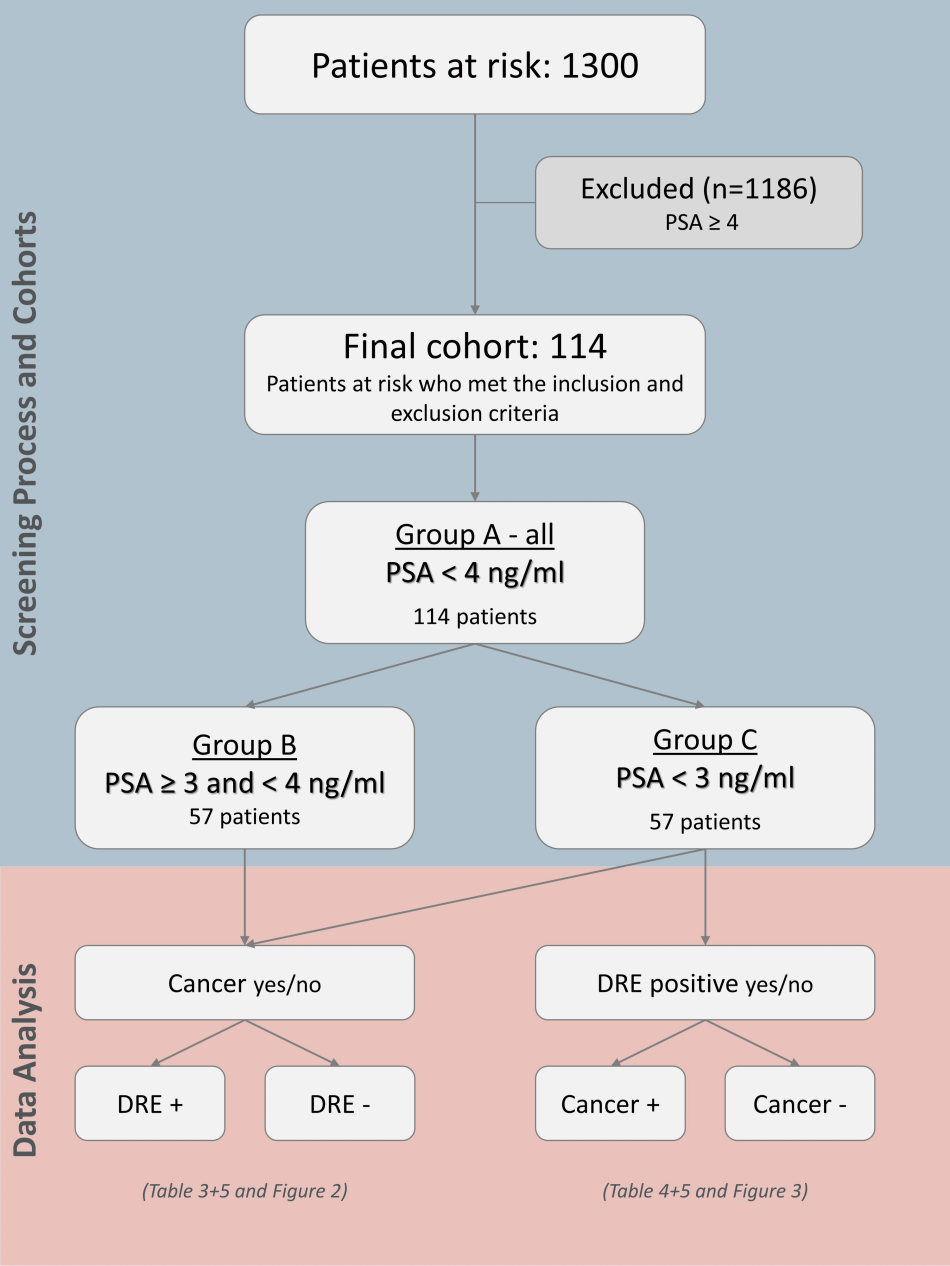
A CONSORT-style flow diagram illustrating patient selection, patients excluded, those fulfilling the inclusion and exclusion criteria PSA: prostate-specific antigen; DRE: digital rectal examination; CONSORT: Consolidated Standards of Reporting Trials

From this cohort, all men with PSA < 4 ng/mL were selected for further analysis (Group A). This group was subsequently stratified into two sub-cohorts: one with serum PSA between 3 and 4 ng/mL (Group B) and a more stringent subset with serum PSA < 3 ng/mL (Group C), reflecting threshold levels currently discussed in contemporary guideline recommendations [5-7]. Patient characteristics are summarized in Table 1. The study selection process is illustrated in the Consolidated Standards of Reporting Trials (CONSORT) flow diagram (Figure 1), while the applied inclusion and exclusion criteria are summarized in Table 2.

**Table 1:** Patients characteristics PSA: prostate-specific antigen; DRE: digital rectal examination; PI-RADS: Prostate Imaging–Reporting and Data System

Parameter		All patients	Group A	Group B	Group C	p-value (B vs. C)	t-test (B vs. C)	F-value (B vs. C)
PSA level		all	PSA < 4 ng/mL	PSA 3-4 ng/mL	PSA < 3 ng/mL			
Size of the cohort		n = 1300	n = 114 (8.8 %)	n = 57 (4.4 %)	n = 57 (4.4 %)			
Age in years mean (SD)		65.8 (± 7.5)	65.27 (± 9.1)	63.1 (± 9.6)	67.44 (± 8.13)	0.011	-2,6	6.76
PSA in ng/mL mean (SD)		9.3 (± 7.8)	2.68 (±0.97)	3.51 (± 0.32)	1.85 (± 0.62)	< 0.001	17.9	320.4
PSA density in ng/mL^2^ mean (SD)		0.08 (± 1.05)	0.08 (±0.04)	0.09 (±0.04)	0.06 (± 0.03)	< 0.001	4.5	20.25
Prostate volume in mL mean (SD)		56.1 (± 29.9)	42.26 (± 22.4)	45.6 (± 25.4)	38.9 (± 18.62)	0.11	1.6	2.56
Number of previous biopsies mean (SD)		0.82 (± 1.05)	0.72 (± 0.9)	0.79 (± 1.06)	0.65 (± 0.69)	0.41	0.84	0.7
Parameter		All patients	Group A	Group B	Group C	p-value (B vs. C)	Chi²-test (B vs. C)	Cramér’s V
DRE positive (n, %)		160 (12.3 %)	28 (24.6 %)	6 (11 %)	22 (38.6 %)	0.0009	11.03	0.31
PI-RADS category (n, %)	3	588 (45.2)	65 (57.0)	34 (59.6)	31 (54.4)	0.799	0.065	0.02
	4	554 (42.6)	38 (33.3)	16 (28.1)	22 (38.6)	0.41	0.68	0.08
	5	158 (12.2)	11 (7.0)	7 (12.2)	4 (7.0)	0.54	0.38	0.06

**Table 2:** Inclusion and exclusion criteria PSA: prostate-specific antigen

Inclusion criteria (all must be fulfilled):
Male patients aged ≥ 18 years undergoing clinical evaluation for suspected prostate cancer
PSA < 4 ng/mL
Availability of a pre-biopsy serum PSA measurement within 3 months before fusion biopsy
Eligibility and consent for MRI–ultrasound fusion-guided biopsy
Written informed consent to participate in the study and for data analysis
Exclusion criteria (excluded if any of the following criteria applied):
PSA ≥ 4 ng/ml
Prior definitive prostate treatment (e.g., radical prostatectomy, external beam radiotherapy, brachytherapy, cryoablation, HIFU, focal laser ablation)
Ongoing androgen deprivation therapy or prior systemic therapy for prostate disease
Incomplete clinical or imaging data (e.g., missing PSA)
Severe comorbidities precluding biopsy under local or general anesthesia
Inability to provide written informed consent

Extracted variables included age, prostate volume, PSA density, and number of prior biopsies. Histopathological assessment followed the International Society of Urological Pathology (ISUP) PCa grade groups. csPCa was defined as ISUP grade group ≥ 2 (corresponding to Gleason score ≥ 7a = 3+4) [12].

Data processing followed the legal regulations stipulated by the Saxon Hospital Act (§29 Sächsisches Krankenhausgesetz), allowing the scientific use of clinical patients. The study was approved by the institutional review board of the University of Leipzig (Ethics Committee reference 280/25-ek; approval date: 19 August 2025).

Statistical analysis

Patients with a history of definitive PCa treatment, including external beam radiotherapy, brachytherapy, cryotherapy, high-intensity focused ultrasound (HIFU), or MRI-guided focal laser ablation, were excluded from the analysis. Additionally, cases with incomplete documentation of serum PSA or prostate volume were removed.

The analysis was conducted in two complementary ways. First, all patients in whom a carcinoma was detected were assessed to determine whether the DRE findings were suspicious or not (Figure 1). Second, all patients with suspicious DRE findings were evaluated to identify whether carcinoma was detected in their biopsies (Figure 1).

Clinical, radiologic, and histopathological parameters were summarized using descriptive statistics. Data management and analysis were performed using Microsoft Excel (Microsoft Corporation, Redmond, WA) and DATAtab (Seiersberg, Austria). Continuous variables are presented as means with standard deviation (SD), while categorical variables are expressed as absolute frequencies and percentages.

Continuous variables were analyzed using the independent samples t-test. Categorical variables were compared using the chi-square test or Fisher’s exact test, depending on expected cell frequencies. Statistical testing was restricted to the comparison between Group B (PSA 3-<4 ng/mL) and Group C (PSA < 3 ng/mL), as Group A (PSA < 4 ng/mL) is not an independent cohort but a composite of both subgroups. Statistical significance was evaluated using two-sided hypothesis tests with thresholds set at p < 0.05 and p < 0.01.

## Results

Overview

A total of 1,300 men who underwent MRI-ultrasound fusion-guided biopsy were included in the analysis. Among these, 114 patients had a serum PSA level < 4 ng/mL (Group A), 57 of them had PSA < 3 ng/mL (Group C), and 57 were in between (Group B) (Table 1).

The three PSA-defined cohorts were comparable with respect to age, prostate volume, and prior biopsy history, indicating that baseline anatomical and diagnostic characteristics did not differ systematically between groups. As expected based on the cohort stratification, both serum PSA and PSA density were lower in the PSA < 3 ng/mL group. Accordingly, the cohorts differ only in PSA-related parameters and not in glandular morphology or diagnostic preselection (Table 1).

PCa detection independent of DRE findings in patients With PSA < 4 ng/mL

**Figure 2:**
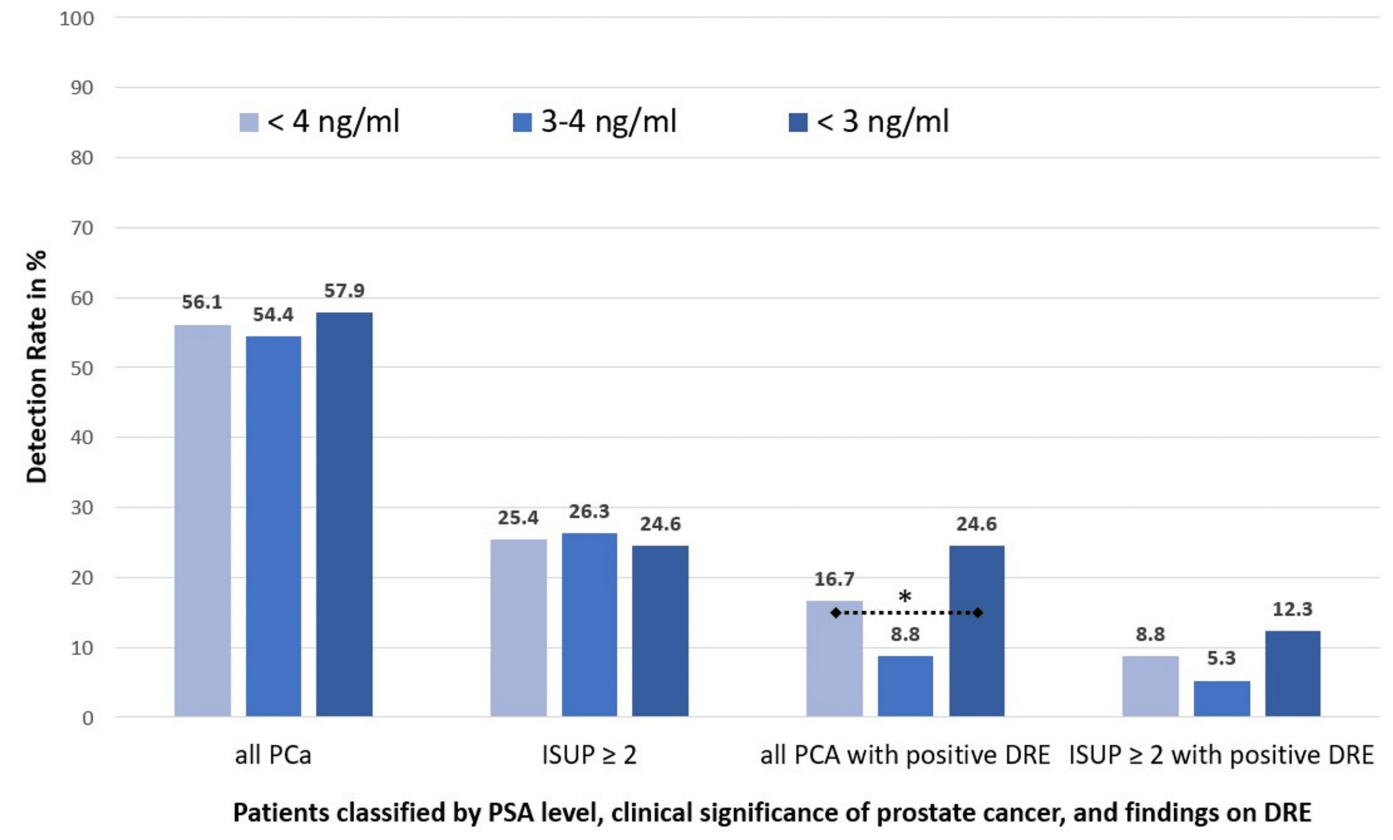
Detection rates of prostate cancer in patients with PSA < 4 ng/ml: primary analysis by cancer presence and secondary analysis by DRE findings, stratified by PSA subgroup * < 0.05; PCa: prostate cancer; PSA: prostate-specific antigen; DRE: digital rectal examination; ISUP: International Society of Urological Pathology

There were no statistically significant differences in overall PCa detection rates among the three groups (56.1% (64/114) vs. 54.4% (31/57) vs. 57.9% (33/57); p = 0.85). Similarly, the detection of csPCa (ISUP ≥ 2) did not differ significantly (25.4% (29/114) vs. 26.3% (15/57) vs. 24.6% (14/57); p = 1.00) (Figure 2).

However, PCas associated with a positive DRE were significantly more frequent in patients with PSA < 3 ng/mL compared to those with PSA 3-4 ng/mL (24.6% vs. 8.8%; p = 0.044). No significant difference was observed in the subgroup of patients with clinically significant cancers who had a positive DRE (p = 0.32) (Table 3 and Figure 2).

**Table 3:** Detection rates of prostate cancer in patients with PSA < 4 ng/ml: primary analysis by cancer presence and secondary analysis by DRE findings PSA: prostate-specific antigen; DRE: digital rectal examination

Parameter	Group A	Group B	Group C	p-value (B vs. C)	Chi²-test (B vs. C)	Cramér’s V (B vs. C)
	PSA < 4 ng/mL	PSA 3-4 ng/mL	PSA < 3 ng/mL			
Size of the cohort	n = 114	n = 57	n = 57			
Prostate cancer, all n (%)	64 (56.1)	31 (54.4)	33 (57.9)	0.85	0.036	0.018
Prostate cancer, ≥ ISUP 2 n (%)	29 (25.4)	15 (26.3)	14 (24.6)	1.0	0.00	0.00
Prostate cancer, all, with positive DRE n (%)	19 (16.7)	5 (8.8)	14 (24.6)	0.044	4.04	0.19
Prostate cancer, ≥ ISUP 2, with positive DRE n (%)	10 (8.8)	3 (5.3)	7 (12.3)	0.32	0.99	0.09

PCa detection in DRE-positive patients only

**Figure 3:**
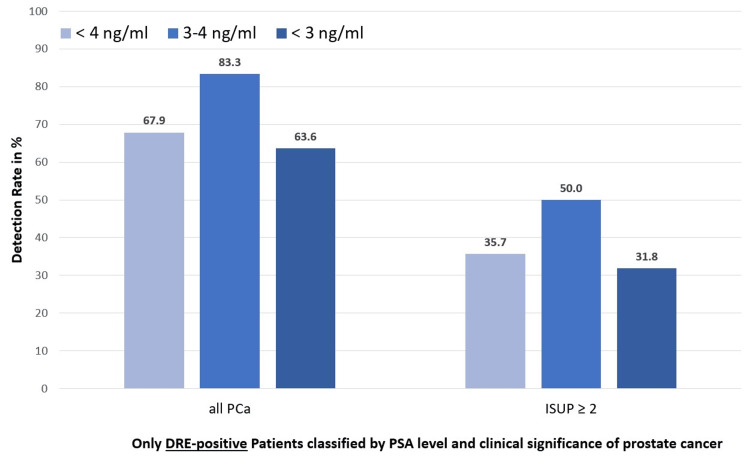
Detection rates of prostate cancer in DRE-positive patients with PSA < 4 ng/mL, stratified by PSA subgroup PCa: prostate cancer; PSA: prostate-specific antigen; DRE: digital rectal examination; ISUP: International Society of Urological Pathology

**Figure 4:**
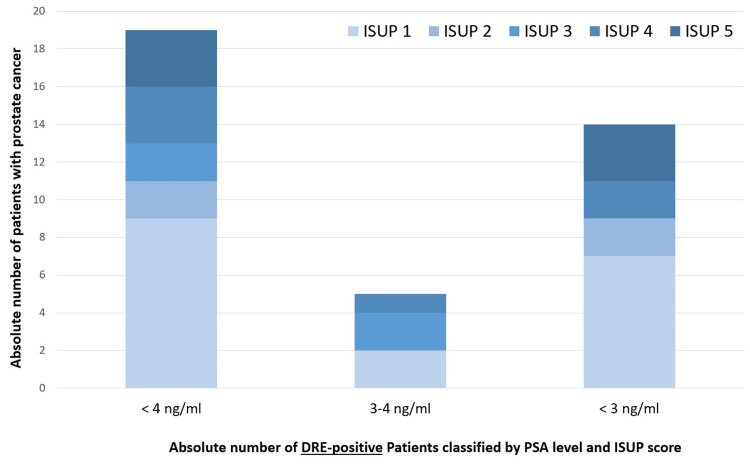
Distribution of ISUP grade groups among DRE-positive patients with PSA < 4 ng/mL, stratified by the PSA subgroup PSA: prostate-specific antigen; DRE: digital rectal examination; ISUP: International Society of Urological Pathology

This group comprises all patients who were detected solely on the basis of a positive DRE and who, in the absence of DRE at that time point, would have escaped further diagnostic work-up within the screening pathway. Among DRE-suspicious patients with PSA < 4 ng/mL, the overall PCa detection rate was 67.9% (19/28), with clinically significant cancer (ISUP ≥ 2) detected in 35.7% (10/28) of cases. Stratification by PSA level showed no significant differences between patients with PSA 3-4 ng/mL and those with PSA < 3 ng/mL (overall cancer detection: 83.3% vs. 63.6%, p = 0.67; clinically significant cancer: 50.0% vs. 31.8%, p = 0.73) (Tables 3-5; Figures 3, 4).

**Table 4:** Detection rates, only DRE-suspicious patients with PSA < 4 ng/mL, stratified by PSA subgroup PSA: prostate-specific antigen; DRE: digital rectal examination

Parameter	Group A	Group B	Group C	p-value (B vs. C)	Chi²-test (B vs. C)	Cramér’s V (B vs. C)
	PSA < 4 ng/mL	PSA 3-4 ng/mL	PSA < 3 ng/mL			
Size of the cohort, n	n = 28	n = 6	n = 22			
Prostate cancer, all n (%)	19 (67.9)	5 (83.3)	14 (63.6)	0.67	0.18	0.08
Prostate cancer, ≥ ISUP 2 n (%)	10 (35.7)	3 (50.0)	7 (31.8)	0.73	0.12	0.07

**Table 5:** Structured data analysis based on the data-analysis component of the CONSORT-style flow diagram, illustrating both evaluation pathways Upper section: First, all patients in whom a carcinoma was detected were assessed to determine whether the DRE was suspicious. Lower section: Second, all patients with suspicious DRE findings were evaluated to determine whether carcinoma was detected on biopsy. PSA: prostate-specific antigen; DRE: digital rectal examination; cs PCa: clinically significant prostate cancer; CONSORT: Consolidated Standards of Reporting Trials

Analysis sequence	First criterion	Second criterion	Group A	Group B	Group C	p-value (B vs. C)	Chi²-test (B vs. C)	Cramér’s V (B vs. C)
			PSA < 4 ng/mL	PSA 3 to < 4ng/mL	PSA < 3 ng/mL			
			n = 114	n = 57	n = 57			
First criterion: Histology; Second criterion: DRE	cs PCa	DRE +	10/114 (8.8%)	3/57 (5.3%)	7/57 (12.3%)	0.32	0.99	0.09
		DRE -	19/114 (16.7%)	12/57 (21.1%)	7/57 (12.3%)	0.32	1.01	0.09
	non-cs PCa	DRE +	9/114 (7.9%)	2/57 (3.5%)	7/57 (12.3%)	0.17	1.93	0.13
		DRE -	26(114 (22.8%)	14/57 (24.6%)	12/57 (21.1%)	0.82	0.05	0.02
	non-PCa	DRE +	9/114 (7.9%)	1/57 (1.8%)	8/57 (14.0%)	0.037	4.34	0.19
		DRE -	41/114 (36.0%)	25/57 (43.9%)	16/57 (28.1%)	0.079	3.09	0.17
First criterion: DRE; Second criterion: Histology	DRE+	cs PCa	10/114 (8.8%)	3/57 (5.3%)	7/57 (12.3%)	0.32	0.99	0.09
		non-cs PCa	9/114 (7.9%)	2/57 (3.5%)	7/57 (12.3%)	0.16	1.93	0.13
		non-PCa	9/114 (7.9%)	1/57 (1.8%)	8/57 (14.0%)	0.032	4.34	0.19
	DRE-	cs PCa	19/114 (16.7%)	12/57 (21.1%)	7/57 (12.3%)	0.32	1.01	0.09
		non-cs PCa	26/114 (22.8%)	14/57 (24.6%)	12/57 (21.1%)	0.82	0.05	0.02
		non-PCa	41/114 (36.0%)	25/57 (43.9%)	16/57 (28.1%)	0.118	3.09	0.17

When considered within the entire cohort of 114 patients, these cancers represent those that would have been missed if DRE had not been performed in the screening setting. Specifically, among patients with PSA < 4 ng/mL, 19/114 (16.7%) PCas and 10/114 (8.8%) clinically significant cancers would have remained undetected without DRE.

In addition, all results were visualized as a structured data analysis based on the data-analysis component of the CONSORT-style flow diagram, illustrating both evaluation pathways. First, all patients in whom a carcinoma was detected were assessed to determine whether the DRE was suspicious. Second, all patients with suspicious DRE findings were evaluated to determine whether carcinoma was detected on biopsy (PCa yes/no → DRE +/- and DRE +/- → PCa yes/no) (Table 5).

Histopathology

ISUP/Gleason score distribution peaked at ISUP 1 (n = 18), while clinically significant cancers (ISUP ≥ 2) were comparatively infrequent (Figure 4).

## Discussion

Summary of our own results and integration into the current literature

Among 1,300 men undergoing MRI-ultrasound fusion biopsy, 114 had a PSA < 4 ng/mL, evenly split into PSA 3-4 ng/mL and PSA < 3 ng/mL subgroups (n = 57 each). Both cohorts were comparable in all baseline characteristics except PSA and PSA density. No significant differences in overall or csPCa (ISUP ≥ 2) were observed between the two PSA strata. However, cancers associated with a positive DRE were significantly more frequent in the PSA < 3 ng/mL group (24.6% vs. 8.8%, p = 0.044), while the detection of clinically significant cancers by DRE did not differ. Among DRE-positive patients, overall and clinically significant cancer detection rates were high and similar across PSA strata. Most tumors were low grade (peaking at ISUP 1), whereas clinically significant cancers were less common.

To address the central question of this study, namely, whether and, if so, which cancers would be missed if DRE were the data, indicates that 8.8% (10/114) of csPCa in patients with a PSA < 4 ng/mL would not be detected.

Our findings align with the established literature demonstrating only modest reliability of DRE, particularly in the setting of low PSA levels. In our cohort, the diagnostic yield of DRE was limited, and only a small number of PCas were detected through palpation alone.

Multiple studies have demonstrated the limited sensitivity of DRE in population-level screening [9,13], and the Cochrane review by Ilic et al. reported no reduction in PCa-specific mortality associated with its use [14]. Consequently, recent guidelines no longer recommend routine DRE for screening purposes but instead emphasize its selective application in specific clinical scenarios [5-7].

Our findings are consistent with this evidence: while DRE alone is insufficient as a screening tool, it may serve as a clinically relevant trigger for further diagnostic evaluation, such as mpMRI or biopsy in selected patients, particularly those with low PSA values but suspicious findings.

Significance of DRE in the modern diagnostic context

The DRE served for decades as a foundational element in the early detection of PCa. Early studies demonstrated its usefulness in combination with PSA testing to improve detection rates of tumors that were clinically palpable [15,16]. However, accumulating evidence has revealed important limitations: DRE primarily detects tumors located in the posterior peripheral zone, requires a minimum lesion volume of approximately 0.2-0.5 cm³ to be palpable, exhibits substantial interobserver variability, and demonstrates sensitivity frequently below 50% even in clinically significant disease [14,17,18].

The introduction of PSA testing fundamentally reshaped PCa diagnostics, enabling the detection of tumors not identifiable by physical examination. Yet, PSA is organ-specific rather than cancer-specific, and elevations may result from benign prostatic hyperplasia, inflammation, or infection, limiting its positive predictive value [19]. Furthermore, clinically significant tumors may exist even when PSA levels are ≤4.0 ng/mL, challenging the reliability of PSA-based thresholds [20].

With the widespread adoption of mpMRI, diagnostic accuracy has increased substantially, particularly for csPCa [21-25]. Consequently, the role of DRE has shifted from primary screening toward a more selective use as part of individualized risk assessment and staging.

In current clinical practice, guidelines increasingly describe DRE as an adjunctive assessment reserved for cases with elevated PSA, abnormal imaging, or clinical suspicion [5-7]. Nonetheless, DRE may retain value in distinct subsets of patients, particularly those with palpable or posteriorly located tumors.

Diagnostic utility of DRE in the setting of low PSA levels

Multiple analyses indicate that the diagnostic yield of DRE is markedly limited at low PSA concentrations. In European screening cohorts, the positive predictive value of DRE in men with PSA <4 ng/mL was as low as 4%-11% [26], and the sensitivity in PSA ranges of 3.0-3.9 ng/mL reached only 11%-20% [27]. Meta-analytic evidence confirms a clinically significant cancer detection rate of approximately 1% using DRE alone, compared with substantially higher rates for PSA-based strategies and mpMRI-driven diagnostic pathways [10,14,19].

Several biological and methodological explanations account for this limited yield: reduced prevalence of palpable tumors at low PSA levels, anatomical inaccessibility of anterior lesions, and the increasing role of quantitative predictive models that outperform subjective tactile examination [19,28]. While combining DRE with PSA marginally increases cancer detection, the incremental value is small [14,19].

Nevertheless, certain clinically relevant tumors are still detected by DRE alone. Approximately 18% of PCas have historically been identified solely based on suspicious DRE findings, including among men with PSA ≤4.0 ng/mL, where positive predictive values range between 5%-30% [22]. In these cases, DRE-positive cancers tend to exhibit higher Gleason scores and greater tumor volumes, suggesting that DRE may disproportionately detect biologically aggressive disease [29,30].

Clinical relevance of abnormal DRE findings despite low PSA Levels

Observational and cohort studies show that DRE-positive cancers in men with low PSA levels are more frequently associated with clinically significant pathology. DRE-detected tumors often demonstrate higher ISUP grade groups, larger volumes, and increased rates of extracapsular extension, indicating prognostic and staging relevance [31-33]. This aligns with clinical experience that some men with PSA <3 ng/mL harbor high-grade tumors that may otherwise evade early detection.

Our own cohort data support this interpretation: while only a small number of cancers were detected via DRE, the few identified cases demonstrated a disproportionately high likelihood of clinical significance. Thus, although the overall diagnostic contribution of DRE at low PSA is small, its case-level yield may be meaningful.

Interobserver variability and limitations of DRE: implications for clinical utility

A central limitation of the DRE lies in its considerable interobserver variability, which affects both diagnostic accuracy and reproducibility across clinical settings. Multiple studies consistently demonstrate that agreement between examiners ranges only from low to moderate, with kappa (κ) values typically between 0.3 and 0.5, even among experienced urologists. This was shown in seminal observational analyses, where palpation-based assessments of prostate consistency, nodularity, and asymmetry varied substantially between examiners [34,35]. These findings underscore the inherently subjective and investigator-dependent nature of DRE.

Additional evidence comes from a large cohort study by Varenhorst et al., who examined 933 men and found complete agreement between a urologist and a general practitioner in only 46.5% of cases. While κ values were moderate for key variables such as size, induration, and nodularity (0.48-0.68), other features, particularly fixation, lateral sulci, and seminal vesicles, showed poor concordance. This work illustrates that even basic palpatory descriptors vary substantially between trained clinicians, reinforcing the structural limitations of DRE as a subjective diagnostic tool [36].

Historical work further illustrates the instability of DRE-based assessment in screening contexts. Large primary care cohorts demonstrated that household physicians and urologists frequently disagreed on what constitutes a suspicious gland, reflecting differences in training exposure and palpation familiarity. Even in specialist settings, studies documented only 38-60% agreement in DRE-based clinical staging, with significant misclassification of cT2 versus cT3 disease [32,33]. This variability directly impacts pre-treatment risk stratification, where overstaging may lead to unnecessary imaging and anxiety, whereas understaging risks delayed or insufficient treatment.

More recent longitudinal data from randomized screening cohorts further highlight how DRE reliability varies with disease prevalence and patient phenotype. In the Finnish Randomized Study of Screening for Prostate Cancer (FinRSPC) and the European Randomized Study of Screening for Prostate Cancer (ERSPC) analyses, DRE performance deteriorated most significantly at low PSA levels [22,27]. Here, examiner disagreement not only increased but also directly contributed to false-positive referrals, increased biopsy rates, and reduced screening efficiency. Conversely, in cases where DRE was positive at low PSA, findings often corresponded to higher Gleason grade and tumor volume [29,30], indicating that while overall yield is low, the few positive cases tend to be clinically significant. This paradox, low sensitivity but high case-level specificity when clearly abnormal, helps explain why professional societies have shifted DRE away from population screening but preserved it in selective diagnostic and staging contexts.

Attempts to improve reproducibility demonstrate partial success. Studies introducing standardized evaluation criteria, structured tactile training, or rating checklists achieved notably higher κ values (up to 0.6-0.8) for individual palpatory features, but these improvements did not translate meaningfully into better cancer detection rates [35]. Similarly, risk calculators (e.g., the Rotterdam Prostate Cancer Risk Calculator) reduce the clinical impact of examiner variability by integrating DRE as only one weighted component, effectively buffering subjective error.

Taken together, the evidence supports a risk-adapted role for DRE. The test performs poorly as a primary screening tool, largely due to limited sensitivity for small or anteriorly located tumors, high investigator dependence, and low predictive value at low PSA levels. Yet, in the presence of a clearly abnormal finding, particularly induration, focal asymmetry, or extracapsular extension, DRE provides diagnostically and prognostically meaningful information that may alter staging (cT3), influence biopsy strategy, or prompt expedited imaging.

Technological new approaches to standardizing and objectifying the DRE

Efforts to reduce examiner-dependent variability in DRE have led to the development of several sensor-based and training-oriented approaches. Recent developments in sensor-based palpation technology, including mechanical and tactile imaging systems, have demonstrated the feasibility of objectively mapping prostate tissue elasticity and geometry. Such devices may reduce investigator variability and support standardized documentation of DRE findings, addressing one of the key methodological limitations of manual palpation. However, most available studies are more than a decade old, and none of these technologies has yet achieved widespread adoption in routine urological practice [36-42]. More recent engineering advances illustrate renewed interest in this field. The E-finger prototype described by Good et al. provides direct elasticity assessment using a micro-sensor capable of quantifying tissue stiffness with high intra-observer reliability (intraclass correlation coefficient (ICC) ≈ 0.85) in cadaveric prostatectomy specimens, demonstrating that small malignant or fibrotic foci may theoretically be detectable through quantitative palpation [40]. Complementary tactile resonance approaches, such as the sensor system evaluated by Åstrand et al., further confirm that mechanically distinct lesions can be detected by analyzing frequency-dependent stiffness signatures, although detection remains limited for dorsally located or deeply embedded tumors [41]. Alongside sensor innovation, human-factor research has highlighted that palpation proficiency itself can be optimized. Simulator-based studies using high-fidelity prostate models showed that specific finger trajectories and pressure-modulation patterns significantly improve detection of small nodules near clinical limits, suggesting that structured simulator training could enhance DRE sensitivity and reduce interobserver variation [42].

Building on these earlier foundations, the recently introduced ProstaPalp™ system by IntelliPalp Dx seeks to transform DRE from a subjective examination into a reproducible, data-driven procedure [43,44]. Using embedded mechanical sensors and algorithm-based signal processing, ProstaPalp™ records pressure variations generated during palpation and derives quantitative indicators of prostate stiffness and asymmetry from these measurements. Early engineering reports indicate potential value for standardization and clinical documentation, though large-scale clinical validation is still pending [45].

Collectively, these technologies exemplify the ongoing effort to shift DRE from a variable, operator-dependent maneuver to a standardized, sensor-supported diagnostic modality capable of producing reproducible, quantifiable data.

Clinical utility of DRE beyond urology

DRE remains a clinically valuable component of physical assessment not only within urology but also across broader medical specialties, owing to its favorable safety profile, minimal cost, and capacity to identify relevant comorbid or incidental findings.

First, when performed correctly, DRE imposes minimal patient burden and carries an exceedingly low risk of adverse events, reinforcing its role as a safe, low-threshold component of routine examination. Reported complications are rare and typically limited to transient discomfort, whereas clinically significant harm is virtually absent in the literature. This robust safety profile supports its continued integration into standard clinical practice.

Second, beyond its established utility in prostate assessment, DRE enables direct palpation of the anal canal and distal rectum, thereby facilitating early identification of anorectal malignancies, particularly when red-flag findings such as a firm mass or blood on the glove are present. A recent case series illustrated this diagnostic potential by documenting a case in which DRE enabled the timely detection of rectal cancer in a younger patient initially presumed to have hemorrhoids [46]. Moreover, the presence of blood or mucus on the examining finger may indicate non-urological pathology, including colorectal neoplasia or other intestinal malignancies, thus serving as an important clinical warning sign during routine evaluation.

Third, DRE aids in the diagnosis of benign anorectal conditions such as hemorrhoids, fissures, and prolapse, which may account for patient symptoms and benefit from early management. Prompt recognition of these conditions can improve patient care and prevent unnecessary delays in treatment. A gastroenterological review highlighted that DRE, when combined with careful perianal inspection, enables early identification of hemorrhoids and fistulas and may reduce the need for more invasive or resource-intensive diagnostic procedures [46,47].

Finally, from a healthcare-systems perspective, incorporating DRE into routine clinical encounters may enhance oncological vigilance in primary and specialist care. Although DRE is not recommended as a stand-alone screening tool for prostate or colorectal cancer due to limited sensitivity, it remains a valuable adjunct when applied judiciously in symptomatic individuals or high-risk groups [48,49]. In summary, DRE is a safe, inexpensive, and widely accessible examination method capable of identifying clinically relevant pathology beyond the prostate. Its judicious application contributes to comprehensive patient assessment and may support earlier detection of both malignant and benign anorectal disease.

Perspective: integration into modern diagnostic pathways and the future role of DRE

Modern PCa diagnostics increasingly favor risk-adapted, imaging-first algorithms, integrating PSA density, MRI-based risk stratification, and clinical nomograms [5-7,21,25]. Current AUA and EAU guidelines do not recommend DRE as a routine screening modality but position it as an optional adjunct in cases of clinical suspicion or when physical findings could influence management [5,6].

However, DRE remains valuable in clinical staging (e.g., detecting extracapsular extension, T3 tumors), biopsy planning when physical findings and imaging are discordant, evaluating symptomatic patients or men with fluctuating PSA, and identifying select high-grade palpable tumors in PSA-low populations.

Future developments may include AI-assisted haptic sensor technologies and structured scoring systems to improve reliability and clinical integration.

In summary, while DRE no longer plays a primary role in PCa screening, it retains diagnostic and prognostic significance in selected clinical contexts, especially where PSA and imaging findings are discordant or where palpation reveals a suspicious lesion.

Limitations

Several limitations of this study should be acknowledged. First, its retrospective, single-center design may limit external validity, as diagnostic thresholds and biopsy indications may differ across institutions. Second, interobserver variability in the performance and interpretation of the DRE cannot be fully controlled, even with cross-checks by two urologists, and the study lacks standardized documentation of palpatory findings (e.g., firmness, nodularity, focal asymmetry), which may influence diagnostic yield. Third, the exclusive inclusion of patients referred for mpMRI-guided fusion biopsy introduces an MRI-era selection bias, as the cohort represents a highly preselected population with elevated pre-test probability. Consequently, our findings may not be generalizable to unselected screening populations or primary care settings where MRI is not routinely performed.

Finally, the relatively small number of cases identified solely by DRE limits the precision of subgroup estimates and the robustness of conclusions regarding this particular patient subset.

## Conclusions

In the era of PSA-based risk stratification and mpMRI-guided diagnostics, the DRE has largely lost its former role as a primary screening tool, and our data confirm its limited overall diagnostic yield in men with PSA < 4 ng/mL. Only a small proportion of cancers in this cohort were detected solely on the basis of an abnormal DRE finding, and most of these were low-grade.

Nevertheless, in patients with distinctly suspicious palpatory findings, the likelihood of detecting PCa, particularly clinically significant disease, remained meaningful across the low PSA range. This underscores that an experienced clinician can gain substantial diagnostic insight from careful observation and physical examination of the individual patient and that DRE, while no longer a stand-alone test, continues to serve as a safe, low-cost, selective assessment capable of identifying cases potentially missed by purely PSA- or imaging-driven pathways.
